# Development and evaluation of a calcium alginate based oral ceftriaxone sodium formulation

**DOI:** 10.1007/s40204-016-0051-9

**Published:** 2016-07-20

**Authors:** Nachiket Patel, Darshan Lalwani, Steven Gollmer, Elisha Injeti, Youssef Sari, Jerry Nesamony

**Affiliations:** 1 Industrial Pharmacy Division, Department of Pharmacy Practice, College of Pharmacy and Pharmaceutical Sciences, The University of Toledo, 3000 Arlington Avenue, MS1013, Toledo, OH USA; 2 Department of Science and Mathematics, Cedarville University, 251 N Main St., Cedarville, OH USA; 3 Department of Pharmaceutical Sciences, School of Pharmacy, Cedarville University, 251 N Main St., Cedarville, OH USA; 4 Department of Pharmacology, College of Pharmacy and Pharmaceutical Sciences, University of Toledo, 3000 Arlington Avenue, MS1013, Toledo, OH USA

**Keywords:** Ionotropic gelation, Calcium alginate, Ceftriaxone sodium, Thermogravimetric analysis, Atomic force microscopy, Controlled release

## Abstract

The purpose of this work was to develop a multiparticulate system exploiting the pH-sensitive property and biodegradability of calcium alginate beads for intestinal delivery of ceftriaxone sodium (CS). CS was entrapped in beads made of sodium alginate and sodium carboxymethylcellulose (CMC), acacia, HPMC K4M and HPMC K15M as drug release modifiers. Beads were prepared using calcium chloride as a cross-linking agent, followed by enteric coating with cellulose acetate phthalate (CAP). The beads were then evaluated for entrapment efficiency using HPLC, in vitro drug release examined in simulated gastric fluid (pH 1.2) and simulated intestinal fluid (pH 6.8), swellability, particle size and surface characterization using optical microscopy, scanning electron microscopy (SEM), and atomic force microscopy (AFM). Thermal gravimetric analysis (TGA) was utilized to check the polymer matrix strength and thermal stability. The drug entrapment efficiency of the optimized formulation was determined to be 75 ± 5 %. Swelling properties of drug-loaded beads were found to be in a range of 0.9–3.4. Alginate beads coated with CAP and containing CMC as a second polymer exhibited sustained release. The drug release followed first-order kinetics via non-Fickian diffusion and erosion mechanism. The particle size of the beads was between 1.04 ± 0.20 and 2.15 ± 0.36 mm. TGA, AFM, and SEM data showed composition and polymer-dependent variations in cross-linking, thermal stability, surface structure, morphology, and roughness. The physico-chemical properties of the developed formulation indicate suitability of the formulation to deliver CS orally.

## Introduction

The oral route of administration is the most convenient, preferable, and desirable method of administering therapeutic agents for systemic effects. Traditional oral formulation methods employed in a majority of currently commercially available pharmaceutical products provide clinically effective therapy and safety to the patient by maintaining the required balance between pharmacodynamic and pharmacokinetic profiles (Brahma and Kwon (2013). To overcome the limitations of conventional immediate-release dosage forms, a wide variety of oral drug delivery systems have been developed in recent years which provide sustained-release dosing and are able to maintain steady plasma drug levels for extended periods of time with reduced side effects (Chein 1992).

Ceftriaxone was the drug of choice in this formulation development since studies from our lab and substantiated by other researchers have demonstrated that intraperitoneal (i.p.) injections of this drug in rats attenuated cue-induced cocaine relapse-like behavior (Sari et al. 2009; Knackstedt et al. 2010). This effect is partly due to upregulation of expression of a major glutamate transporter, which is called glutamate transporter 1 (GLT-1), in several brain regions. Several subsequent studies revealed that ceftriaxone treatment upregulated GLT-1 expression and consequently reduced alcohol consumption in male and female alcohol-preferring rats (Sari et al. 2006, 2011, 2013). Additionally, it was found that ceftriaxone-attenuated, relapse-like alcohol-drinking behavior was associated with upregulation of GLT-1 and GLT-1 isoforms (GLT-1a and GLT-1b) in male alcohol-preferring rats (Alhaddad et al. 2014; Qrunfleh et al. 2013; Rao and Sari 2014).

Despite promising preclinical results to potentially treat drug dependence, including alcohol and cocaine, with ceftriaxone, its clinical use as therapeutic for drug addiction is limited because it can only be administered IV or IM. This is due to the fact that ceftriaxone sodium, a third-generation cephalosporin is highly water-soluble but cannot be absorbed orally due to its acid labile nature and poor permeability across the GI epithelia. To overcome this situation, the drug must be incorporated into delivery systems that provide greater stability in the gastric fluids and also release the dose in a sustained manner (Lee et al. 2005).

Multiparticulate delivery systems are often used to obtain controlled drug delivery, enhance drug stability and improve the bioavailability of acid labile drugs. The potential advantage of a multiparticulate system includes reduced risk of local irritation, reduced risk of dose dumping, increased bioavailability and less inter- and intrasubject variability (Roy and Shahiwala 2009). Multiparticulate beads have been prepared via ionotropic gelation technique by cross-linking polyelectrolyte biopolymers such as alginates, sodium carboxymethylcellulose, and hydroxypropylmethylcellulose to counter ions such as calcium or aluminum to produce insoluble meshwork in the form of beads that would provide the sustained drug release (Narra et al. 2012; Sanli et al. 2007; Patil et al. 2010). Sodium alginate was chosen as the biopolymer for this research because alginates are naturally occurring anionic polysaccharides that are biodegradable, non-toxic, and have high biological safety and ability to incorporate acid labile drugs into the matrix formed after cross-linking (Almeida and Almeida 2004).

Sodium Alginate is a hydrophilic biopolymer and naturally occurring polysaccharide obtained from brown seaweed and algae (George and Abraham 2006). It has been used widely as a controlled-release excipient due to its biodegradable nature. It has a unique property of gelation in aqueous media in the presence of multivalent ions such as calcium and aluminum that leads to the formation of a unique egg box-like gel structure. This property makes it possible to encapsulate both macromolecular agents and low molecular weight therapeutic agents within the polymer matrix. The hydrogel forming property also allows sodium alginate preparations to release an encapsulated drug in a controlled-release manner, dependent on the pH of the liquid medium in which it comes into contact (Deasy 1984).

A multiparticulate system was developed and evaluated for the intestinal delivery of ceftriaxone sodium by ionotropic gelation technique using sodium alginate as the hydrophilic carrier. Sodium carboxymethylcellulose, acacia, HPMC K4M and HPMC K15 were used as drug release modifiers in various proportions. Cellulose acetate phthalate, a pH-sensitive polymer was used to sustain the release, prevent drug degradation in gastric fluids, and deliver ceftriaxone sodium specifically to the intestine. The mixture of the drug and polymer dispersion was added drop wise into aqueous calcium chloride solution, resulting in instantaneous gelation to form beads. The beads were evaluated for entrapment efficiency, loss on drying, particle size, swelling index, and in vitro drug release. The morphology and surface of the beads were characterized by scanning electron microscopy (SEM) and atomic force microscopy (AFM).

## Materials and methods

### Materials

Ceftriaxone sodium was obtained from Apotex Corp. (Weston, FL). Sodium alginate (medium viscosity) was purchased from Sigma-Aldrich, (St. Louis, MO). Sodium carboxymethylcellulose (viscosity 7MF) was purchased from Amend Drug and Chemical Co. (Irvington, NJ). Acacia was obtained from PCCA (Houston, TX). Hydroxypropylmethylcellulose (HPMC) K4M Premium CR and K15M Premium CR were purchased from the Dow Chemical Company (Midland, MI). Calcium chloride (anhydrous) was purchased from Fischer Scientific (Fair Lawn, NJ) and cellulose acetate phthalate was purchased from Spectrum Quality Products, Inc. (New Brunswick, NJ). 2.06M Tetrabutylammonium Hydroxide (TBAOH) was purchased from Thermo Fischer Scientific (Sunnyvale, CA). All other solvents and reagents used were of analytical grade.

## Methods

### Preparation of beads

Multiparticulate beads of ceftriaxone sodium were prepared using ionotropic gelation technique. Initially, four separate sets of beads were prepared using varying concentrations of sodium alginate alone. Subsequently, four sets of coated and uncoated beads were prepared using sodium alginate in combination with polymers such as sodium carboxymethylcellulose, acacia, HPMC K4M and HPMC K15M. Calcium chloride was used as a cross-linking agent. The detailed composition of various formulations is described in Table 1. The composition of the polymer beads was optimized by modifying parameters such as drug-polymer ratio, combination with other polymers, concentration of cross-linking agent, entrapment efficiency, curing time and in vitro drug release data.

**Table 1:** Composition of beads

Batch code	Sodium alginate (% w/v)	Calcium chloride (% w/v)	Sodium carboxymethyl cellulose (% w/v)	Acacia (%w/v)	HPMC K4M (% w/v)	HPMC K15M (% w/v)	Cellulose acetate phthalate (% w/v)
Formulations with alginate alone							
F1	0.4	2	–	–	–	–	–
F2	1	2	–	–	–	–	–
F3	1	1	–	–	–	–	–
F4	0.5	0.4	–	–	–	–	–
Uncoated formulations with alginate and polymer blends							
F5	1.25	3.75	4.25	–	–	–	–
F6	1.25	3.75	–	2.5	–	–	–
F7	1.25	3.75	–	–	1.875	–	–
F8	1.25	3.75	–	–	–	1.25	–
Coated formulations with alginate and polymer blends							
F9	1.25	3.75	4.25	–	–	–	5
F10	1.25	3.75	–	2.5	–	–	5
F11	1.25	3.75	–	–	1.875	–	5
F12	1.25	3.75	–	–	–	1.25	5

Each formulation contains 2 mg of ceftriaxone sodium. Blank (without drug) formulations were also prepared for formulations F1–F12

**Fig. 1:**
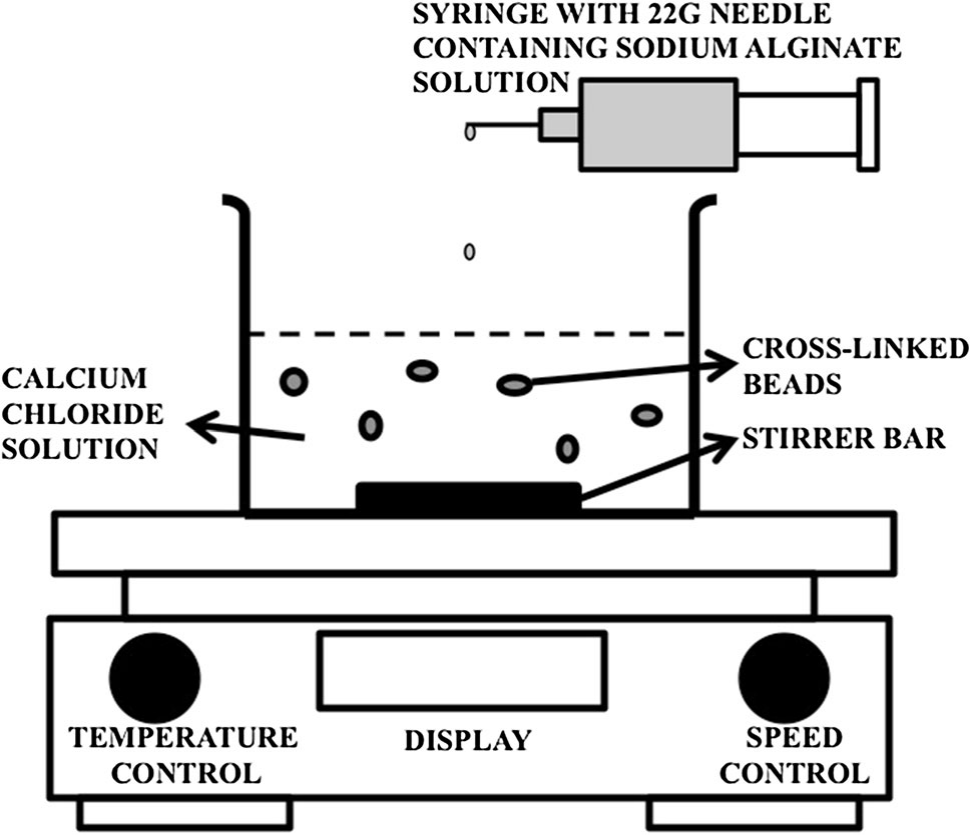
Schematic diagram of the preparation of beads by ionotropic gelation method

Various concentrations of sodium alginate 0.4–1 % (w/v) were prepared by dissolving the polymer in deionized water at room temperature for 2 h under magnetic stirring at a speed of 1200 rpm. 2 mg of ceftriaxone sodium was dissolved in the sodium alginate solution and was then stirred for another 30 min for homogenous mixing of the drug-polymer solution. The solution was sonicated for 15 min in an ultrasonicating water bath to remove air bubbles that were formed during the drug-polymer mixing. Bubble-free drug-polymer solution was then introduced drop wise via a syringe attached with a 22-guage hypodermic needle into varying concentrations of aqueous calcium chloride solution (cross-linking agent) under gentle agitation at room temperature, as shown in Fig. 1. The concentrations of calcium chloride ranged from 0.2 to 4 % (w/v). The droplets from the drug-polymer solution instantaneously gelled upon contact with the calcium chloride solution (Das and Senapati 2007). The drug-loaded beads were allowed to cure for another 15 min to enhance the rigidity of the beads. The beads were then recovered by filtering through a filter paper (Whatman no. 42), spread on petri dish, dried for 2 days at room temperature and stored in an air-tight container for further use.

Beads were also prepared using polymers mixed with sodium alginate such as acacia, sodium carboxymethylcellulose, HPMC K4M and HPMC K15M as shown Table 1. These polymers were added after dissolving the drug in aqueous sodium alginate and were stirred for 4 h at 125–350 rpm to ensure content uniformity.

### Preparation variables and process optimization

Variables such as concentration of sodium alginate, concentration of calcium chloride, concentration of acacia, concentration of sodium carboxymethylcellulose, concentration of HPMC K4M, concentration of HPMC K15M, curing time and stirring speed were evaluated during optimization of the bead preparation process (Manjanna et al. 2009). All the prepared beads were analyzed for shape, size, drug loading, percent yield, entrapment efficiency and in vitro drug release. On the basis of the results obtained, the process parameters were optimized as follows: Sodium alginate concentration: 1.25 % w/v, sodium carboxymethylcellulose concentration: 4.25 % w/v, acacia concentration: 2.5 % w/v, HPMC K4M concentration: 1.875 % w/v, HPMC K15M concentration: 1.25 % w/v, calcium chloride concentration: 3.75 % w/v, curing time: 15 min, and curing speed: 60 rpm. The optimized process parameters were used for preparing all further batches, including preparation of coated beads.

### Coating of beads

Formulations F5, F6, F7 and F8 were selected for enteric coating with cellulose acetate phthalate as they showed desirable shape, size, and good entrapment efficiency. Cellulose acetate phthalate is a pH-sensitive polymer that dissolves in aqueous medium when the pH is above 6.2. The enteric coating solution was prepared using cellulose acetate phthalate (5 % w/v) in 1:1 mixture of ethyl acetate and ethanol. This solvent system completely dissolves cellulose acetate phthalate while maintaining the integrity of the calcium alginate beads. Beads from the optimized batches F9, F10, F11 and F12 were dispersed in the enteric coating solution and then stirred at 1200 rpm for 30 min at room temperature. The coated beads were harvested by filtering it with filter paper, spread on petri dish and dried for 2 days at room temperature and stored in an air-tight container for further use.

### Analysis of ceftriaxone sodium by RP-HPLC

Ceftriaxone sodium was analyzed using reversed phase-high-performance liquid chromatography (RP-HPLC) (Waters Alliance e2695 separation module, Milford, MA) equipped with a Phenomenex C18 column (Gemini 5u, 110A, 250 × 4.6 mm) and photodiode array detector (Waters Alliance 2998). The aqueous mobile phase consisted of 2.06 M tetrabutylammonium hydroxide 5.26 % (v/v) (pH 7.0, adjusted with o-phosphoric acid) and acetonitrile (ACN) in a ratio of 70:30 (v/v), respectively (Shrivastava et al. 2009). Isocratic separation method was utilized with a flow rate of 0.7 ml/min and column temperature set to 25 °C. The injection volume was 10 µl with a run time of 10 min. The retention time of ceftriaxone sodium was found to be 7.3 min with an absorption wavelength (*λ*_max_) of 240 nm. Various calibration standards of ceftriaxone sodium were prepared in deionized water by serial dilution ranging from 100 to 0.39 µg/ml. Each standard was quantitatively analyzed (*n* = 3) and the average peak area was plotted against concentration to prepare a calibration curve.

### Determination of percent yield, drug loading and entrapment efficiency

Approximately 200 mg of beads was completely dispersed in 50 ml of phosphate buffer solution (pH 7.4) and stirred for 3 h at a speed of 1200 rpm. After stirring, an aliquot from the solution was taken in a 2 ml Eppendorf tube and centrifuged (Eppendorf centrifuge 5430R, UK) for 5 min at 14,000 rpm to remove any insoluble solids. The supernatant was then analyzed for the drug content using HPLC. Observations were recorded in triplicate and percentage yield, drug loading and entrapment efficiency were calculated using the following formulas:

$$ {\text{\%  Yield}} = \frac{\text{Total weight of beads}}{{{\text{Total weight of polymers}} + {\text{drug}}}} \times 100 $$

$$ {\text{\%  Drug loading}} = \frac{\text{Actual drug weight}}{\text{Total weight of beads}} \times 100 $$

$$ {\text{Drug entrapment efficiency}} = \frac{\text{Actual drug content}}{\text{Theoretical drug content}} \times 100 $$

### Thermal gravimetric analysis (TGA)

TGA was performed to evaluate the strength of the polymer matrix and thermal stability by recording phase transitions and degradation patterns of various blank formulations (without drug) based on the percent weight loss and decomposition (Devi and Kakati 2013). TGA was performed by utilizing thermal analysis instrument (TA Q50, USA) under nitrogen gas. Platinum pan sample holder was initially tared, and accurately weighed amounts of beads were transferred to it. The dynamic nitrogen flow rate was 20 ml/min, and the heating rate was 10 °C/min with a temperature range of 25–650 °C.

### Swelling index study

This study was conducted to estimate the percentage swelling of the beads that causes leaching or degradation of the drug in the gastric fluid. Only batches with an entrapment efficiency of more than 30 % were selected for further studies. Dried ionically, cross-linked beads increase their volume after few minutes in water or in buffers due to matrix rehydration that is dependent on the degree of cross-linking (Crcarevska et al. 2008). 200 mg of beads were suspended in 50 ml of simulated gastric fluid (pH 1.2) and samples were shaken at 60 rpm speed in a mechanical shaker (Thermo Scientific™ Precision reciprocating shaker bath, USA) and allowed to swell for 2 h at 37 ± 0.5 °C, simulating the gastric medium. After 2 h, the beads were carefully removed, blotted dry and weighed. The difference between the initial and final weights of the beads was used to determine water sorption, and the swelling index was calculated using the following formula,

$$ {\text{Swelling index}} = \frac{{W_{\text{f}} - W_{\text{o}}  }}{{W_{\text{O}} }} $$

where *W*_o_ is the initial weight of beads and *W*_f_ is the final weight of the beads after swelling.

### Atomic force microscopy (AFM)

The AFM studies were done in a Nanosurf Easyscan 2 AFM instrument (Nanosurf AG, Switzerland) using an NCLR cantilever. The samples were analyzed in the dynamic force-tapping mode. In this mode, the instrument chooses the optimal vibration frequency to be used during measurements. The frequency range that is available for use in this mode is 15–500 kHz. The instrument was tested for XY calibration using a silicon substrate grid containing an array of silicon oxide squares of height 100 nm separated by 10 μm. The AFM data were utilized to determine the surface morphology and coating of various drug-loaded and blank beads. Parameters such as surface structure, morphology, and roughness were evaluated for the blank (without drug), drug-loaded uncoated beads, and drug-loaded coated beads (Rokstad et al. 2014). Randomly selected beads were placed on a double-sided tape fixed on an AFM stub when the tests were done. The position of the cantilever above the particle surface was visually ascertained before lowering the cantilever to just above the bead. Later, the automatic approach mode was enabled that let the cantilever slowly hover to the sample surface. The collected data were analyzed in the accompanying easyScan 2 software.

### Determination of water content in alginate beads

Calcium alginate beads from F5, F6, F7 and F8 preparation batches were weighed immediately after preparation and later after being subjected to drying. The mean water loss from the beads was calculated from the following equation,

$$ W_{\text{l}}\, \% = \, [(W_{\text{o}} - W_{\text{d}} )/W_{\text{o}} ] \times 100 $$

where *W*_0_ is the initial weight before drying and *W*_d_ represents the final weight after drying.

### Particle size analysis

An optical microscope (Nikon SMZ800, USA) fitted with an ocular and stage micrometer, having accuracy of 0.01 mm, was used to determine the particle size of the beads. Analysis of the prepared beads was performed using a resolution of 30× to determine the diameter of 10 randomly selected beads. The instrument was calibrated at 1 unit of eyepiece micrometer equal to 1/30 mm (33.33 μm), and the average diameter of the beads was calculated using the following equation,

$$ X = \frac{{\mathop \sum \nolimits (X_{\text{i}} )}}{N} $$

where, *X* = average particle diameter, *X*_i_ = individual diameter of beads and *N* = number of beads. In vitro drug release study

In vitro drug release studies were performed using a mechanical shaker (Thermo Scientific™ Precision reciprocating shaker bath, USA) at 37 ± 0.5 °C and 60 rpm. The beads of formulations F9, F10, F11 and F12 were placed in 50 ml of enzyme-free simulated gastric fluid (pH 1.2) for the first 2 h, followed by enzyme-free simulated intestinal fluid (pH 6.8). 5 ml aliquots of the dissolution fluid was withdrawn at regular time intervals and replaced with an identical volume of dissolution media. The aliquots from various time intervals were then centrifuged (Eppendorf centrifuge 5430R, UK) for 15 min at 14000 rpm to remove insoluble solids, and the supernatant was analyzed for drug content using a HPLC at a wavelength (*λ*_max_) of 240 nm. The tests were performed in triplicate, and the cumulative percentage of ceftriaxone sodium released was calculated using a regression equation generated from the calibration curve.

### Analysis of release kinetics and mechanism

The in vitro drug release data from the dissolution study were analyzed by fitting various kinetic models such as zero-order (% release vs. time), first-order (log % retained vs. time), Higuchi (% release vs. square root of time) and Korsmeyer-Peppas equation. Correlation coefficient values were obtained by regression analysis of the plots.

### Loose surface crystal study (LSC)

The purpose of this study was to estimate the amount of un-entrapped drug present on the surface of the beads. Approximately 200 mg of beads from formulations F9, F10, F11 and F12 were suspended in 50 ml of phosphate buffer (pH 6.8), and samples were shaken vigorously at 200 rpm for 15 min in a mechanical shaker (Thermo Scientific™ Precision reciprocating shaker bath, USA) with the temperature set to 37 ± 0.5 °C. The amount of drug dissolving from the surface of the beads was analyzed at 240 nm using HPLC, and the percentage of drug released with respect to entrapped drug in the sample was recorded (Manjanna et al. 2009).

### Scanning electron microscopy (SEM)

SEM was utilized to study the external morphology (size, shape and surface) of the prepared beads. Randomly selected beads were placed on double-sided copper conductive tape (NEM Nisshin EM Co. Ltd.) fixed on aluminum stubs. The beads were then sputter-coated with a thin layer of gold in a vacuum for 45 s at 20 mA using a coating unit (Cressington 108 auto sputter coater, UK) to make it electrically conductive and was analyzed with a SEM instrument (FEI Quanta 3D FEG Dual Beam Electron Microscope, USA) operated at 5 kV.

## Results and discussion

### Analysis of ceftriaxone sodium by RP-HPLC

**Fig. 2:**
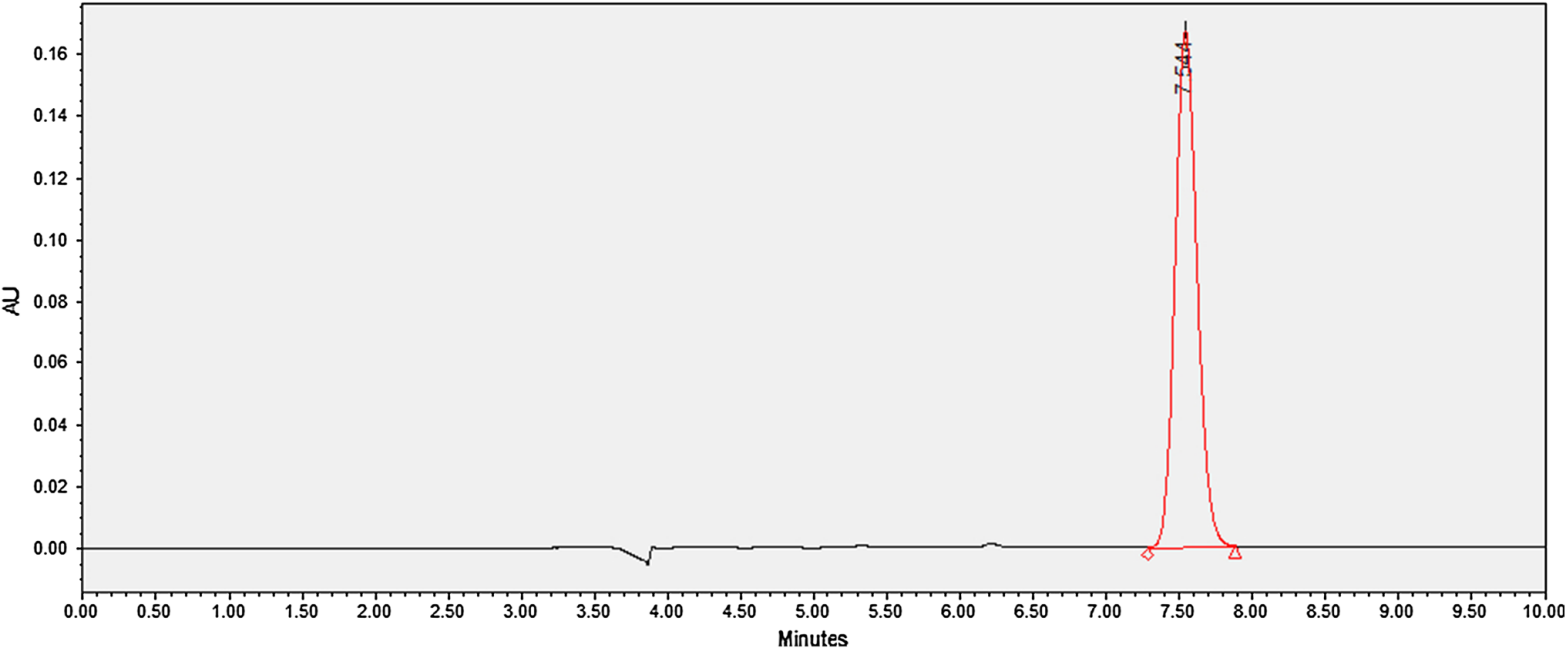
Representative HPLC chromatogram of ceftriaxone sodium

From the chromatogram shown in Fig. 2, it can be observed that the ceftriaxone sodium peak was successfully separated and eluted with no tailing at a retention time of 7.3 min. To make certain that highly polar drugs such as ceftriaxone are retained in the HPLC column, an ion-pairing agent has to be added to the mobile phase (Trautmann and Haefelfinger 1981). Tetrabutylammonium hydroxide (TBAOH) was used as the ion-pairing reagent since it was reported to allow separation of ionic and highly polar substances during HPLC (Trautmann and Haefelfinger 1981). Various other flow rates were used when developing the analytical method, but the flow rate of 0.7 ml/min gave a favorable signal to noise ratio, with adequate separation time. HPLC method validation for ceftriaxone sodium was performed according to ICH guidelines and found to be linear in the range of 50–0.78 µg/ml with a correlation coefficient of 1. This value of correlation coefficient indicates that there was negligible random error, and good linearity was associated with the method. This further confirms the goodness of fit of the peak area versus ceftriaxone concentration data to the linear equation. The regression equation obtained from the calibration curve was *y* = 35167*x* + 209, where *y* is the peak area and *x* is the drug concentration. The small sample volume of 10 µl and the run time of 10 min allows rapid analysis of ceftriaxone during routine laboratory use. The selectivity of the HPLC method was determined when no deviation in the baseline was seen when an analytical placebo (containing all other components present in the sample except ceftriaxone sodium) was injected during an HPLC run. The percentage recovery of ceftriaxone sodium ranged from 99.37 to 103.32 %, and intra-day precision was found to be 0.49–0.88 % (measured using  %RSD) (FDA 2000). The high recovery values indicate that other components used in the sample and the HPLC analysis had no adverse effects on the quantitative determination of ceftriaxone sodium.

### Determination of percent yield, drug loading and entrapment efficiency

The entrapment efficiency for the formulations F1–F4 was found to be in the range of 8.2 ± 1.5 to 14.8 ± 1.8 %. Such low drug entrapment efficiency may be attributed to leakage of the drug into the cross-linking solution due to high porosity and low density of the alginate-polymer matrix (Sankalia et al. 2005). This is a severe limitation that leads to drug loss during preparation of alginate beads using ionotropic gelation and has been reported by other researchers (Anal et al. 2006; Pongjanyakul and Rongthong 2010). Since a viable formulation cannot be prepared with such low entrapment efficiency, several studies were performed to increase the drug entrapment efficiency. Initially, sodium alginate and calcium chloride concentrations were methodically modulated when beads were prepared, as these parameters were reported to enhance drug loading and impart controlled-release characteristics (Al-Musa et al. 1999; Blandino et al. 1999). When this approach was investigated, the observed ceftriaxone entrapment efficiencies ranged from 3.6 to 17.6 %. This indicated that ceftriaxone entrapment efficiency improved marginally with changes in the polymer and calcium chloride concentrations. Since it has been reported that gelation of alginate is instantaneous with rapid expulsion of water from the gel matrix in acidic conditions (Yotsuyanagi et al. 1991), organic acids including acetic acid, citric acid, fumaric acid, maleic acid, and tartaric acid were used to adjust the pH of the calcium chloride solution between 2 and 6. The organic acids were added to the calcium chloride solution and pH was verified using a pH meter. Sodium alginate containing ceftriaxone sodium was extruded into the acidic calcium chloride solution, and entrapment efficiency was evaluated (Table 2). With this method, the ceftriaxone entrapment efficiencies ranged between 11 and 23.9 %. Subsequently, various polymers were mixed with the sodium alginate dispersion before extruding into the calcium chloride solution. A variety of polymers have been used in conjunction with sodium alginate to modulate drug loading and modify drug release kinetics (Pillay and Fassihi 1999; Nochos et al. 2008; Hua et al. 2010). Various polymers were selected based on published literature on the topic and availability of the polymer. These polymers were added to sodium alginate solution and drug loaded beads were prepared and drug entrapment efficiencies were determined. Formulations that demonstrated the highest drug entrapment efficiencies were identified and used in further studies. Additionally, several studies reported benefits of coating calcium alginate beads with polymers such as chitosan, polyacrylamide, and cellulose acetate phthalate (Iskakov et al. 2002; Won et al. 2005; Bodmeier et al. 1989). Hence, entrapment efficiencies for formulations F5 to F12 were optimized using various polymers in combination with sodium alginate and also using cellulose acetate phthalate as the coating polymer. Chitosan was not used in our optimized formulation because it contributed to a significant increase in particle size and agglomeration after recovering and drying the coated beads. Additionally, acidic chitosan dispersion would have to be used as the coating agent, which can further be detrimental to ceftriaxone stability. The entrapment efficiency of the optimized uncoated formulations F5 to F8 were found to be in range of 38.2 ± 1.6 to 67.8 ± 2.4 %, with formulation F5 possessing the highest entrapment and F6 the lowest. The optimized formulations were then coated with cellulose acetate phthalate (F9–F12), and the entrapment increased significantly for all the coated formulations. F9 possessed the highest entrapment (74.7 ± 5.0 %), whereas F10 had the lowest entrapment (40.9 ± 0.7 %) of all coated formulations. The higher entrapment efficiency observed with the coated formulations was further investigated using optical microscopy (Nikon model TiU coupled with Photometric Coolsnap EZ 20 MHz monochrome camera) to investigate how the beads dried (data not shown). Coated and uncoated beads were prepared and immediately placed on glass slides and optical micrographs were obtained and analyzed using the Metamorph software available with the microscope. It was found that water from the uncoated beads seeped out of the beads immediately after preparation but the entrapped water did not gush out from the coated beads. Additionally the coated beads had a brown color as opposed to the clear appearance of uncoated beads after preparation when observed under the microscope indicating the formation of CAP coating as soon as the beads were coated. Thus from the uncoated beads it was concluded that the drug dissolved in the aqueous phase was being lost during the initial bead drying phase rapidly as water gushed out. However, since the water oozing out phenomenon was markedly diminished in CAP-coated beads less amount of the dissolved drug was lost leading to higher entrapment efficiency. The coated beads appeared to dry more through evaporation of the entrapped water. Drug loading was directly proportional to the ratio of the amount of drug used during preparation of beads to the total weight of beads produced (Heller and Himmelstein 1985). Drug loading was found to be low and in a range of 0.07 ± 0.01 to 1.66 ± 1.39 % in all the formulations. Similar results have been reported by other researchers as well (Anal et al. 2006; Whitehead et al. 2000). The reduced drug loading was deemed due to the high porosity of the alginate matrix that allows water-soluble drug to leach out when beads are prepared, recovered and dried. The problem was abated when a co-polymer was mixed with sodium alginate to prepare drug-loaded beads (Whitehead et al. 2000; Fontes et al. 2013; Ishak et al. 2007). The low drug loading in our formulations is also attributed to the small amount of drug (~2 mg) used in the preparation of the trial formulations. This was further confirmed when beads of batch F9 were prepared using a proportionately higher amount of ceftriaxone sodium per gram of the polymer blend.

**Table 2:** Effect of polymer concentration, calcium chloride concentration, and coating polymer concentration on percent yield and entrapment efficiency of various formulations

Batch code	Yield (%)	Drug entrapment efficiency (%±SD, *n* = 3)
F1	24.9	8.2 ± 1.5
F2	64.0	9.9 ± 2.2
F3	68.2	12.8 ± 0.9
F4	66.2	14.8 ± 1.8
F5	90.0	67.8 ± 2.4
F6	35.7	38.2 ± 1.6
F7	47.4	42.8 ± 1.8
F8	42.8	40.5 ± 3.4
F9	93.6	74.7 ± 5.0
F10	45.5	40.9 ± 0.7
F11	64.0	48.1 ± 2.5
F12	69.0	41.1 ± 3.8

In this work a classical approach similar to one-factor-at-a-time method of formulation and selection through trial and error was used when choosing drug-loaded beads for further studies. Initially beads were prepared with just aqueous calcium chloride and aqueous sodium alginate containing a particular amount of dissolved ceftriaxone sodium. The sodium alginate concentration was kept constant and the calcium chloride concentration varied in a series of experiments. Subsequently, the calcium chloride concentration was kept constant and the sodium alginate concentration was varied. In all these experiments the amount of ceftriaxone sodium was kept constant. The drug-loaded beads were dried and entrapment efficiencies were determined. The formulations that showed good entrapment efficiency F1, F2, F3, and F4 are described in detail and shown in this manuscript. Among these the one that showed the highest drug entrapment efficiency was selected for mixing/blending with a second polymer. The second polymer was also added in various amounts and the entrapment efficiencies determined for each combination. Of the four polymer combinations containing beads those that possessed the highest entrapment efficiencies are described and evaluated in detail in the manuscript namely; F5, F6, F7 and F8. Formulations F5, F6, F7, and F8 were then coated and further evaluated. The formulation selected was always based on the highest entrapment efficiency observed in that particular combination of excipients. The primary objective of developing a formulation for orally administering a ceftriaxone dose of 50 mg/kg was thus achieved using sodium alginate-sodium CMC beads coated with CAP.

Percent yield was in the range of 24.9–93.6 %, where F9 possessed the highest percent yield (93.6 %) and F1 possessed the lowest percent yield (24.9 %). When sodium alginate was the sole polymer used in the beads, a better yield was observed when the polymer concentration was higher, as can be seen when comparing the yield of beads F1, F2, F3, and F4. When the sodium alginate and calcium chloride compositions were both 1 % w/v, the highest percentage yield was observed. This phenomenon can be explained by the differences in the degree of cross-linking between the beads. The ability of calcium ions to consolidate the alginate-polymer chains into an egg-box structure is dependent on the diffusivity of the cross-linker solution in the polymer droplet (Tavakol et al. 2013). At a low sodium alginate concentration, the polymer chains on the exposed surface of the droplet that comes into contact with the calcium chloride solution are rapidly cross-linked (Al-Musa et al. 1999). This prevents further diffusion of calcium ions into the extruded polymer droplet, thereby leaving the inner polymer chains un-crosslinked. When the polymer concentration is increased, the surface cross-linking proceeds at a relatively slower rate, enabling penetration of calcium ions into the droplet and thereby, producing better cross-linking of the polymer chains present further away from the surface (Aslani and Kennedy 1996). When sodium CMC, acacia, and two grades of HPMC were mixed with sodium alginate, the yield appeared to vary with the co-polymer that was used. Compared to CMC and the two grades of HPMC, when acacia was used with sodium alginate, the second lowest yield of beads in this study was observed. This is potentially due to the chemical composition of acacia. It is highly heterogeneous, consisting of a range of highly branched molecular structures containing more or less similar monosaccharide compositions (Islam et al. 1997; Street and Anderson 1983). It is possible that sodium alginate, being a linear polymer, is unable to incorporate large amounts of branched acacia structures within the gel matrix when beads are formed, leading to a low yield. Further characterization methods will provide conclusive evidence regarding the degree of interaction between sodium alginate and acacia. Sodium CMC and the two grades of HPMC are linear polymers. However, the difference in the yield between CMC and HPMC is attributed to highly viscous dispersions that were produced when sodium alginate was mixed with HPMC. The thick dispersions reduce diffusivity of the gel matrix, reducing penetration of calcium ions and thereby impacting the cross-linking process and consolidation of the polymer chains during formation of the beads (Siepmann and Peppas 2012; Rajabi-Siahboomi et al. 1996).

### Thermal gravimetric analysis (TGA)

**Fig. 3:**
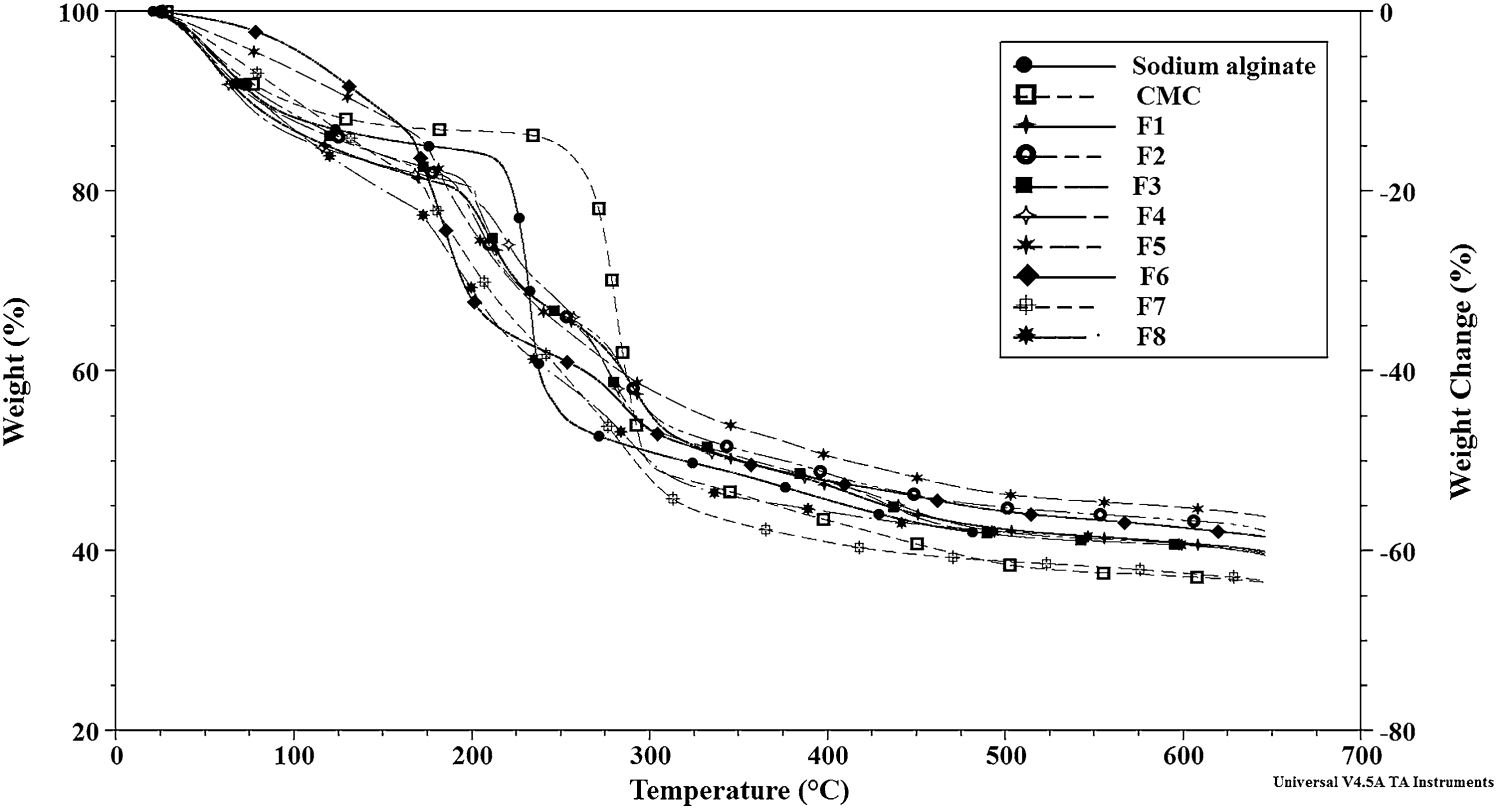
Comparison of TGA thermograms of pure alginic acid, pure CMC and blank bead formulations of F1 to F8

TGA was performed to investigate the degradation and thermal stability of the beads. The thermograms (Fig. 3) show a stepwise and sequential weight-loss pattern. The first thermal event associated with all samples F1, F2, F3, and F4 is attributed to loss of water. This weight loss was observed in the temperature range of 25–170 °C and depended on the nature of the sample. Approximately 17 % weight loss was observed during this event in the calcium alginate beads prepared using various sodium alginate and calcium chloride solutions. The next weight loss of about 15 % was observed in F1, F2, and F3 in the temperature range of approximately 170–250 °C. This weight loss is typically associated with destruction of glycosidic bonds (Soares et al. 1995). Another weight loss of about 15 % was seen in the same samples in the temperature range of 250–370 °C. This weight loss is also related to degradation of glycosidic bonds. At this point, approximately 50 % of the sample still remained and corresponds to intermediate carbonaceous char material. The weight loss of about 6 % seen between 370 and 530 °C in F1, F2, and F3 is associated with oxidation of previously formed intermediate carbonaceous char material. In bead F4, the weight loss observed after the water loss thermal event is much more complex and disparate than the other samples. Between 180 and 205 °C, a loss of 3 % was observed which is substantially lower than that observed in F1, F2, and F3 in the same temperature range. Three sequential weight loss events were observed in F4 as follows: 205–240 °C with a weight loss of 9 %, 240–345 °C with a weight loss of 18 %, and 380–545 °C with a weight loss of 7 %. When comparing drug entrapment efficiency of formulations F1 to F4, it was found that F4 showed the highest drug entrapment. F4 beads were also prepared using the least amount of calcium chloride. Therefore, it can be postulated that the superior thermal stability and better drug entrapment observed in F4 beads is likely from effective cross linking of the polymer matrix due to better diffusion of calcium into the alginate matrix during preparation. Calcium ions form complexes with various functional groups in the alginate-polymer, and other researchers have reported the complexity involved in this process (Fontes et al. 2013). As was found in this work, the polymer gel strength of alginate beads as determined by TGA was reported to depend on the concentration of calcium chloride used during bead preparation, the nature of the ionic salt used, sodium alginate concentration, and the presence of other polymers (Bajpai and Tankhiwale 2006).

Beads prepared using sodium alginate mixed with another polymer yielded qualitatively different TGA results. The thermal events corresponding to calcium alginate beads containing acacia (formulation F6) were as follows: initial weight loss event of about 9 % in the temperature range of 35–135 °C, followed by a weight loss of 27 % between 145 and 240 °C, then a weight loss of 14 % from 240 to 380 °C, and a final loss of 3 % in the 410–540 °C temperature range. Calcium alginate beads containing HPMC K4M (formulation F7) and HPMC K15M (formulation F8) had similar weight loss patterns during the initial parts of the TGA experiment. Both types of beads had nearly similar weight loss in the temperature range 30–160 °C, with F7 beads losing 18 % and F8 beads losing 20 % weight. Similarly, in the temperature range 160–240 °C, F7 and F8 beads lost 18 % weight. In the temperature range of 240–500 °C, when glycosidic bonds undergo pyrolysis, F7 beads lost 24 % weight. However, F8 beads lost weight sequentially in a two-step process, first a weight loss of 14 % between 240 and 380 °C, followed by a 3 % weight loss between 380 and 520 °C. In formulation F5, which contained CMC as the second polymer in the calcium alginate beads, the weight loss was slightly different in nature. There was no weight loss in F5 below 100 °C. The first weight loss event in F5 was of about 12 % in the temperature range of 110–250 °C, followed by a small loss of 7 % between 250 and 300 °C. Following this, there were three sequential and small weight loss events: the first one at 305–360 °C of about 4 %, the second event of 3 % between 360 and 420 °C, and the third loss of 4 % between 420 and 550 °C. A comparison of weight loss events between formulations F5, F6, F7, and F8 shows that the TGA data correlated well with the weight change information obtained from the water loss data reported later in this paper. Additionally, formulation F5 demonstrated smaller weight loss at high temperature ranges during TGA when compared to F6, F7, and F8 beads. This indicates that beads formulated using sodium alginate and CMC had better thermal stability to the pyrolytic effects of high temperature than other formulations.

### Swelling index study

The “swelling-dissolution-erosion” process in alginate hydrogels is highly complex, with the osmotic pressure gradient between the gel and the environment playing an important role in the swelling process. The swelling phenomenon is generally initiated by the relaxation of the polymer network at high osmotic pressure (Pasparakis and Bouropoulos 2006). The swelling ratio of the beads depend on the pH of the solution in which the bead is placed, since swelling of calcium alginate beads under acidic conditions is insignificant (Tønnesen and Karlsen 2002).

The low swelling index (Table 3) of the calcium alginate-polymer beads (F5–F8) in acidic pH is probably due to the proton-calcium ion exchange forming insoluble alginic acid regions as the solvent penetrates into the dense gel network. However, the beads show marginal swelling even in the acidic environment, possibly due to penetration of water through pores in the bead surface causing weight gain from hydration of the hydrophilic groups. The swelling behavior of the coated beads containing additional polymers (F9–F12) was observed to be lower when compared to formulations F5–F8. This is because of the presence of pH-sensitive enteric coating polymer covering the dense polymer matrix of beads. At the pH of SGF (1.2), the CAP molecules remain un-ionized (Raffin et al. 1996). This decreases the swelling by preventing solvent penetration into the gel network. CAP-coated beads also showed some swelling due to solvent entry through cracks on the bead surface that were visible during SEM studies.

**Table 3:** Swelling index of calcium-alginate beads

Batch code	Swelling index
F5	3.4
F6	1.6
F7	3.0
F8	2.4
F9	2.1
F10	0.9
F11	1.8
F12	1.5

For a drug to get released, the alginate beads must absorb solvent and swell significantly. The results suggest that the dried beads can swell slightly in the stomach and later be transferred to the upper intestine where the particles potentially will swell significantly and finally erode into the lower intestine (Manjanna et al. 2009).

### Atomic force microscopy (AFM)

**Fig. 4:**
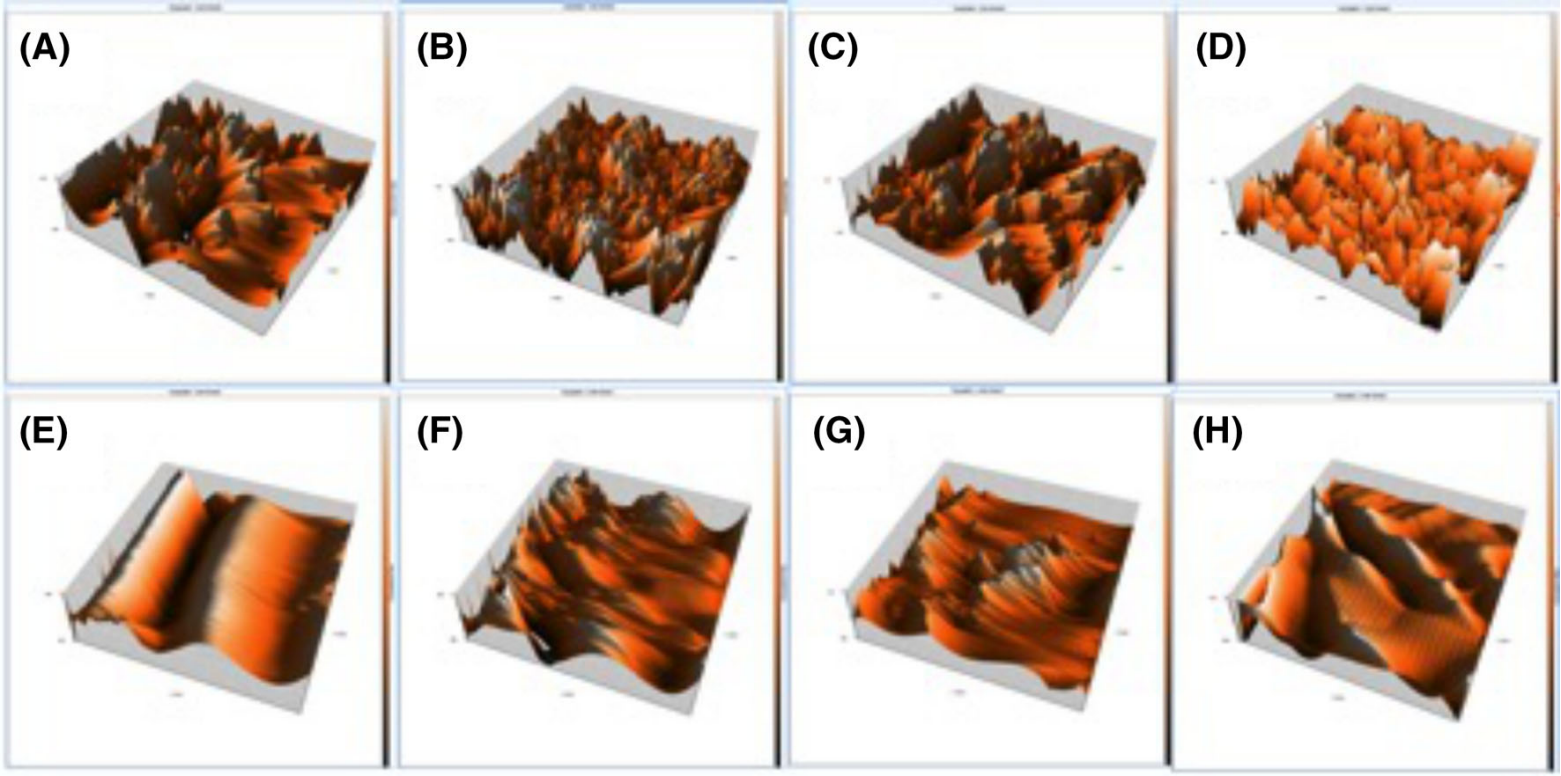
Topographic images of uncoated blank beads (*upper row*) of formulations **a** F5, **b** F6, **c** F7 and **d** F8; Topographic images of coated blank beads (*lower row*) of formulations **e** F9, **f** F10, **g** F11, and **h** F12

**Fig. 5:**
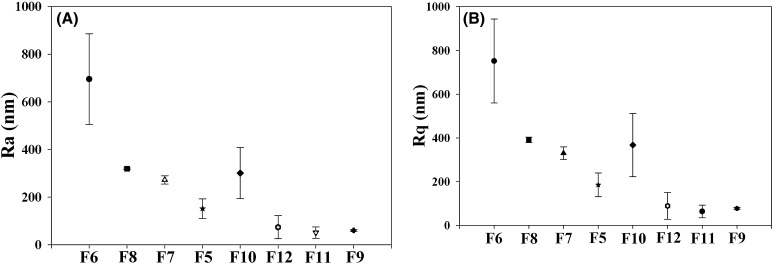
AFM data **a** *R*_*a*_ values of various formulations, **b** *R*_*q*_ values of various formulations

Atomic force microscopy (AFM) was used to obtain information about the topographical features of the beads such as morphology and roughness (Rokstad et al. 2014). The images shown in Fig. 4a–f depict the 3D topographical comparison between the uncoated and coated formulations. The uncoated beads (Fig. 4a–d) have sharp, irregular and spiky projections on the surface, indicating a coarse or rough texture. By contrast, the coated beads (Figs. 4e–h) have smooth-edged projections, surfaces, and grooves. The AFM images indicate that the coating polymer was successfully deposited on the beads during the coating process. To quantify the difference in surface roughness between various samples, two parameters were obtained during data analysis, including roughness average (*R*_a_) (Fig. 5a) and root mean square (RMS) roughness (*R*_q_) (Fig. 5b) (Lungan et al. 2015). The *R*_*a*_ is described as $R_{a} = \frac{1}{L}\int\nolimits_{0}^{L} {\left| {Z(x)} \right|} dx$, where the function *Z*(*x*) describes the surface profile in terms of height “*Z*” and position “*x*” of the sample over the measurement length “*L*”. *R*_*q*_ is defined as $R_{q} = \sqrt {\frac{1}{L}\int\nolimits_{0}^{L} {\left| {Z^{2} (x)} \right|} dx}$. *R*_*q*_ is similar to *R*_*a*_, with the difference that the mean squared absolute value of *Z*(*x*) is used. This enables accounting for the peaks and valleys on a measured surface, since the square of the amplitude is used in the calculations. All samples showed slightly higher values for *R*_*q*_ than *R*_*a*_. The formulation F5 (uncoated calcium alginate-acacia beads) displayed the highest *R*_*a*_ and *R*_*q*_ values. The surface roughness of the uncoated beads based on *R*_*a*_ and *R*_*q*_ values can be arranged in order from highest to lowest as F6 > F8 > F7 > F5. These data corroborate information obtained from the drug entrapment efficiency studies. The lowest drug entrapment efficiency was observed in F6, suggesting that the high roughness value is possibly evidence of the high porosity of the beads. The drug entrapment efficiency of the uncoated formulations followed an identical pattern to that of the surface roughness. Both *R*_*a*_ and *R*_*q*_ values substantially dropped to lower values in all the coated formulations, implying that the coating polymer was deposited on the bead surface after the coating process. The *R*_*a*_ value of formulation F6 decreased by approximately 57 % from 695.6 ± 190.3 to 300.8 ± 107.4 nm after coating (formulation F10). The corresponding decrease in the R_q_ value was by approximately 51 %, from 751.9 ± 191.8 to 367.4 ± 145.1 nm. The surface roughness values (*R*_*a*_ and *R*_*q*_) of the coated beads can be arranged in order from highest to lowest as F10 > F12 > F9 > F11. The 3D topographic images also confirm the variations in Ra and Rq data seen in various formulations.

### Determination of alginate beads’ water content

The results shown in Table 4 reveal that water loss was highest in formulation F8, followed by F7, F5 and F6. Since the concentrations of alginic acid and calcium chloride were identical in all the formulations, the highest amount of water loss in formulation F8 may be attributed to the presence of HPMC K15M due to the polymer’s ability to retain high amounts of water when compared to other polymers (Al-Musa et al. 1999). F6 demonstrated the least water loss, which is due to the rapid drying nature of acacia. F5 demonstrated less water loss than F7 and F8; this could be due to the formation of a dense polymer matrix of sodium alginate and CMC and gelation with a smoother surface and fewer pores. These observations were similar to the weight loss results obtained from TGA studies reported in this study.

**Table 4:** Percent of water loss from calcium-alginate beads

Batch code	Water loss (%)
F5	78.7 ± 1.3
F6	76.7 ± 0.7
F7	82.1 ± 1.7
F8	89.1 ± 1.5

### Particle size analysis

The mean particle size of various formulations (F5–F12) was in the range of 1.04 ± 0.20 and 2.15 ± 0.356 mm (Table 5). Particle size distribution of each formulation was found to be within a narrow range, but the mean particle size varied between different formulations. This could be due to the increase in the relative viscosity at high concentrations of polymers, thus leading to the formation of large droplets during the addition of drug-polymer solution into the calcium chloride solution when the beads were prepared. The results indicated that the coated formulations (F9, F10, F11, and F12) have larger particle sizes compared to the uncoated formulations (F5, F6, F7, and F8). This could be attributed to the accumulation of the coating polymer on the bead surface, resulting in an increase in the diameter of the beads. The slightly larger particle size seen in the coated beads further indicates that the coating polymer was retained on the bead surface, producing a successful coating over the particles.

**Table 5:** Particle size of calcium-alginate beads

Batch code	Particle size (mm ± SD, *n* = 3)
F5	1.36 ± 0.19
F6	1.04 ± 0.20
F7	1.44 ± 0.18
F8	1.49 ± 0.31
F9	1.59 ± 0.15
F10	1.17 ± 0.17
F11	1.80 ± 0.26
F12	2.15 ± 0.36

### In vitro drug release study

**Fig. 6:**
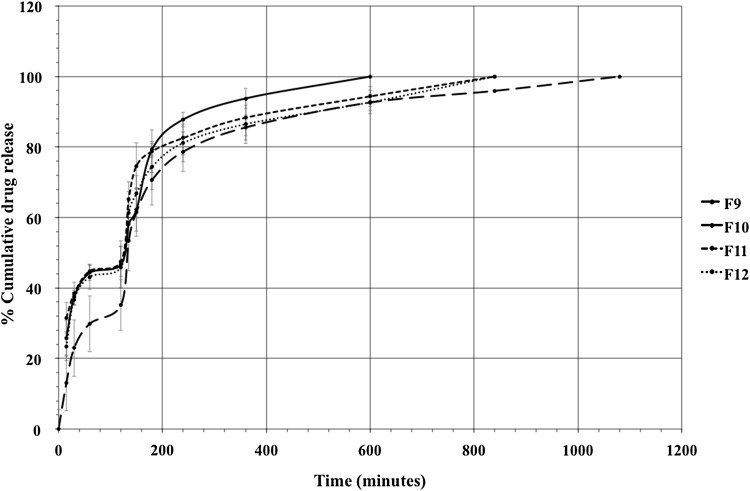
In vitro drug release profile of ceftriaxone sodium-loaded formulations F9, F10, F11 and F12. Results indicate mean % ±SD, (*n* = 3)

The in vitro drug release profile of ceftriaxone sodium in enzyme-free simulated gastric fluid (SGF) followed by enzyme-free simulated intestinal fluid (SIF) from the CAP-coated calcium alginate beads prepared with various sodium alginate-polymer blends is shown in Fig. 6. The drug release from alginate beads is dependent on the penetration of the dissolution medium into the beads, swelling and dissolution of the alginate matrix, and the dissolution of the drug subsequent to leaching through the swollen matrix (Mandal et al. 2010). Within the first 2 h, 35–47 % of the drug was released in SGF from all the coated formulations. This is related to the presence of nicks on the surface of the beads. The nicks occur due to partial collapse of the polymer network via dehydration when coating with organic solvents such as ethyl acetate (Mandal et al. 2010). The remaining part of the entrapped drug was released in the SIF, with sustained release for up to 10 h in F10, 14 h in F11 and F12, and 18 h in F14. This phenomenon is due to the high degree of swelling observed when alginate beads are exposed to liquid media of decreasing acidity and increasing alkalinity. The phosphate anions present in SIF have very strong affinity for calcium ions that hold the alginate-polymer chains together in the cross-linked gel matrix at pH greater than 5 (Dainty et al. 1986). Therefore, calcium alginate beads have been found to disintegrate in SIF within 4 h after initial exposure. Even in the presence of cracks on the bead surface, drug release continued for an extended period of time, probably due to the dense gel network preventing the leaching out of the drug from the beads. Additionally, the beads that were tested in the in vitro release studies after swelling remained intact during the course of the study, with smaller fragments appearing occasionally during the in vitro release experiment. When alginate was combined with other polymers such as pectin, chitosan, CMC and various other polymers before coating with enteric polymers, it was found that the gel strength, robustness, and drug release properties were enhanced when compared to beads prepared using alginate alone (Anal et al. 2006; Kim et al. 2012; Fontes et al. 2013). The more rigid gel formed by the alginate-polymer mixture reduces gel erosion by decreasing the calcium ion diffusion from the cross-linked calcium alginate matrix. When comparing the SGF and SIF in vitro segments, it can be seen that a significant portion of the entrapped drug may be released in the intestine rather than the gastric environment. Similar results were obtained when ampicillin was encapsulated in calcium alginate beads reinforced with chitosan. A small fraction of the entrapped ampicillin was released initially into SGF, and subsequent exposure in SIF led to complete and controlled-release of the drug (Anal et al. 2006).

### Analysis of release kinetics and mechanism

Ceftriaxone is currently not available as an oral formulation. The calcium alginate-polymer blend bead reported here is a new delivery system designed to explore oral delivery of ceftriaxone. Thus, by fitting the in vitro release data to various mathematical models and analyzing the release kinetics, we sought to perceive the underlying mechanisms of drug transport across the swelling gel matrix. This would enable us to make quantitative predictions regarding how the ceftriaxone beads would ultimately release the drug at the intended target. The kinetic models used to fit the in vitro release data were zero-order, first-order, Higuchi, and Korsmeyer-Peppas models. The following equations and descriptions provide the general approach.

*Zero order* $C = C_{\text{o}} - k_{\text{o}} t$ , where *C*_o_ is the initial concentration of drug and *k*_o_ is the zero-order rate constant expressed in units of concentration/time, and *t* is the time in hours. Dosage forms from which a constant drug release occurs over a period of time follows this model.

*First order* Log $C = {\text{Log }}C_{\text{o}} {-}kt/2.303$, where *C*_o_ is the initial concentration of drug and is the first-order constant.

*Higuchi’s model* $Q = kt^{1/2}$, where *k* is the constant reflecting the design variable of the system. Although the original Higuchi equation was developed to describe drug release from thin ointment films, it has been extended to apply in various controlled-release systems and geometries as well.

*Korsmeyer-Peppas model* $Mt/M\infty = k_{\text{kp}} t^{n}$, where *Mt/M∞* is fraction of drug released at time *t*, *k*_kp_ is the rate constant, and *n* is the release exponent. For finding the mechanism of drug release, the first 60 % of drug release data were fitted in the Korsmeyer-Peppas model (Arora et al. 2011). This model was one of the first to consider drug release from a polymer matrix that swells when it comes into contact with water.

The interpretation of data was based on the value of the correlation coefficients, which can be found in Table 6. The goodness of fit for various models in general followed from best to least in the following order: first-order > Korsmeyer-Peppas > Higuchi > Zero-order. From the regression coefficients, it can be concluded that the release kinetics for all the coated formulations (F9–F12) can be best described by the first-order model. The results also indicate that a combination of diffusion- and erosion-based drug release mechanisms may be involved in release kinetics (Peppas 1985).

**Table 6:** In vitro release kinetic parameters of ceftriaxone sodium beads coated with cellulose acetate phthalate

Batch code	Zero order [*R* ^2^]	First order [*R* ^2^]	Higuchi [*R* ^2^]	Korsmeyer-Peppas	
				[*R* ^2^]	‘*n*’ values
F9	0.6824	0.9731	0.8478	0.8816	0.461
F10	0.7801	0.9794	0.9016	0.8724	0.2364
F11	0.7256	0.9464	0.8837	0.8961	0.1613
F12	0.6935	0.9683	0.8537	0.8675	0.1538

Some of the critical assumptions involved in the derivation of Higuchi’s equation include homogenous distribution of drug within the matrix, a constant diffusion coefficient for the drug in the matrix, and the requirement that the matrix cannot swell during drug release. The beads reported in this work are porous, and the drug distribution within the bead matrix may not be entirely homogenous. Additionally, the beads are formed by mixing two polymers and finally coated with a third polymer. Hence, the drug diffusion coefficient may be expected to vary within the polymer matrix as it is released. The enteric polymer coat erodes in slightly alkaline media and the bead matrix undergoes swelling and erosion in SIF. Thus, the drug release from these beads may not conform to Higuchi’s equation. The zero-order model did not fit the in vitro release data since the calcium-alginate/polymer mix beads were found to slowly and gradually undergo erosion during the in vitro release testing. Zero-order drug release has been found to apply to polymer-based drug delivery systems prepared using polymers that slowly dissolve in the dissolution medium. The Korsmeyer-Peppas model used to fit the in vitro release data is consistent with drug release from non-swellable polymeric drug delivery systems of various geometries. Since the alginate-based hydrogel used in the ceftriaxone-loaded beads swell in SIF, the in vitro release data do not comply well with the Korsmeyer-Peppas model. Formulations that swell but erode slowly (Liu and Krishnan 1999) and that are porous (Mulye and Turco 1995) have been shown to exhibit first-order release kinetics. The polymer matrix in the calcium alginate-polymer blend beads is porous, albeit much less when mixed with other polymers, and undergoes slow erosion with swelling. The in vitro release thus appears to fit the first-order model better than other models used to fit the data.

### Loose surface crystal (LSC)

The LSC study was an important parameter to determine the amount of the drug present on or near the surface of the beads. This phenomenon may lead to drug degradation or leaching of the drug in gastric fluids (i.e., at pH 1.2) (Abu-Izza et al. 1996). As seen in Table 7, LSC was highest in the F10 formulation, whereas it was lowest in formulation F11. The in vitro release data of formulations F9 to F12 further confirmed the results obtained from this study, as these beads released ceftriaxone when placed in simulated gastric fluid (pH 1.2) for 2 h. Thus, more drug was present near the surface of these beads, contributing to less entrapment within the beads. Migration of drug from within the polymer matrix to surface layers is a common phenomenon when the solvent leaves the polymer during the drying of the beads. Additionally, the porosity of the beads immediately after preparation may cause diffusion of the drug to the periphery of the polymer particle. Additional layers of polymer chains, when present within the sodium alginate-polymer chains, can produce constraints to accommodate drug molecules within the interior regions of the gel. This can also contribute to migration and deposition of the drug near or onto the surface of the polymer beads.

**Table 7:** Percentage of loose surface crystals of ceftriaxone sodium on calcium-alginate beads

Batch code	Loose surface crystal (%)
F9	28.5 ± 4.2
F10	39.6 ± 2.5
F11	23.8 ± 6.6
F12	34.0 ± 2.3

### Scanning electron microscopy (SEM)

**Fig. 7:**
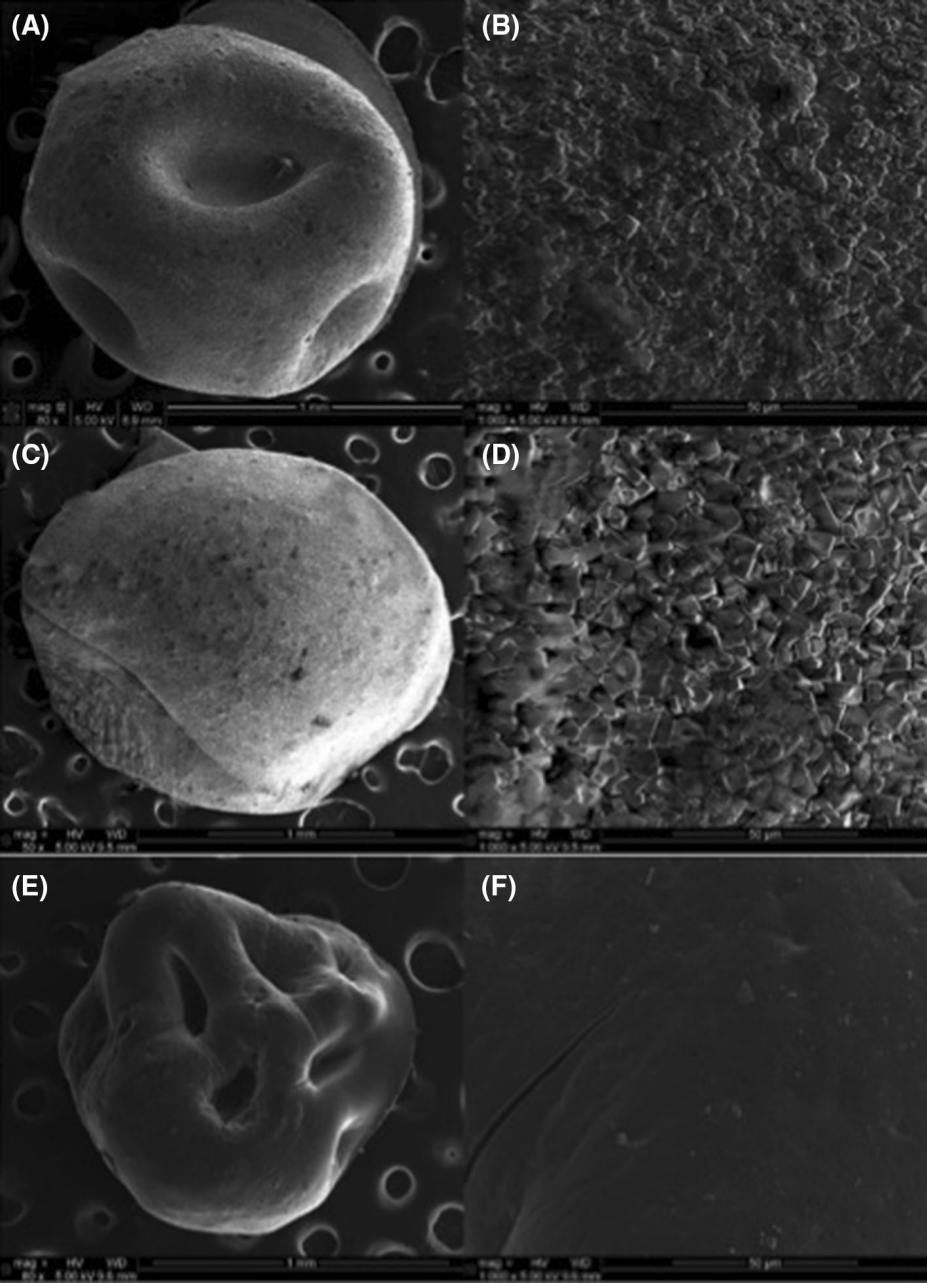
SEM images—blank formulation F5 at **a** low magnification, and **b** at high magnification; **c** drug-loaded formulation F5 low magnification, and **d** high magnification; **e** drug-loaded CAP-coated formulation F9 at low magnification, and **f** high magnification

Based on the entrapment efficiency and in vitro results, formulations F5 and F9 were chosen for the SEM studies. Scanning electron micrographs of blank and drug-loaded beads (F5 & F9) prepared by sodium alginate, sodium carboxymethylcellulose and cellulose acetate phthalate as enteric coating polymer are shown in Fig. 7a–f. SEM micrographs of the blank formulation (Fig. 7a–b) show that the beads were almost spherical, with a rough outer surface. From the images (Fig. 7c–d), F5 beads can be seen to possess a rough surface with a large number of pores that cause the rapid release of drug into the in vitro release medium. It also exhibited a sandy appearance because of the surface-associated crystals of drug (Manjanna et al. 2009). On the other hand, SEM images (Fig. 7e–f) of coated beads (F9) show a smooth surface and possibly a reduced number of pores due to the presence of the coating, which led to a decrease in the drug release rate from beads. However, the surface also exhibits cracks that can be attributed to the partial collapse of the polymer network due to dehydration after coating with organic solvents such as ethyl acetate. This leads to the degradation or leaching of the drug from beads into the gastric fluid, as was seen during in vitro release studies performed in SGF (Mandal et al. 2010). The SEM micrographs of the dry beads also show indentations on the surface, suggesting that the internal structure is porous. This was evident because the beads appeared perfectly spherical immediately after preparation. As the beads dried the surface demonstrated increasing roughness with pits on the surface, demonstrating that large amounts of water from the gelation medium were retained immediately after preparation. As the beads dried, the retained water emanated out through the highly porous polymer, network creating depressions where the local concentration of the pores is largest. This leads to a collapse of the gel network onto the space previously largely occupied by the retained water. The dents on the uncoated beads are less pronounced than the ones observed on coated beads. This is most likely due the rapid evaporation of organic solvents used in the coating solution, leading to a quicker loss of the retained water from the beads.

## Conclusion

It can be concluded that the ionotropic gelation technique can be successfully used for preparation of ceftriaxone sodium beads using sodium alginate in the presence of other polymers such as sodium CMC, HPMC K4M, HPMC K15M and acacia as drug release modifiers. Parameters such as polymer concentration, calcium chloride concentration, and coating polymer concentration played a vital role in achieving high entrapment efficiency, proper particle size, swelling behavior, and surface morphology. Significant differences in the in vitro drug release was observed between various formulations coated using cellulose acetate phthalate. Calcium alginate beads swelled at pH 1.2 but underwent diffusion and erosion at pH 6.8. The use of sodium alginate, carboxymethylcellulose and cellulose acetate phthalate decreased the drug release behavior in gastric conditions to some extent but sustained the drug release at intestinal pH. Further investigation using in vivo models should be performed to substantiate the in vitro results.
